# Generation and characterization of antagonistic anti-human CD39 nanobodies

**DOI:** 10.3389/fimmu.2024.1328306

**Published:** 2024-03-25

**Authors:** Stephan Menzel, Yinghui Duan, Julia Hambach, Birte Albrecht, Dorte Wendt-Cousin, Riekje Winzer, Eva Tolosa, Anne Rissiek, Andreas H. Guse, Friedrich Haag, Tim Magnus, Friedrich Koch-Nolte, Björn Rissiek

**Affiliations:** ^1^Institute of Immunology, University Medical Center Hamburg-Eppendorf, Hamburg, Germany; ^2^Mildred Scheel Cancer Career Center HaTriCS4, University Medical Center Hamburg- Eppendorf, Hamburg, Germany; ^3^Core Facility Nanobodies, University of Bonn, Bonn, Germany; ^4^Department of Neurology, University Medical Centre Hamburg-Eppendorf, Hamburg, Germany; ^5^Cytometry und Cell Sorting Core Unit, Dept. of Stem Cell Transplantation, University Medical Centre Hamburg-Eppendorf, Hamburg, Germany; ^6^Department of Biochemistry and Molecular Cell Biology, University Medical Centre Hamburg-Eppendorf, Hamburg, Germany

**Keywords:** nanobody, CD39, ENTPD1, VHH, single-domain antibody

## Abstract

CD39 is the major enzyme controlling the levels of extracellular adenosine triphosphate (ATP) via the stepwise hydrolysis of ATP to adenosine diphosphate (ADP) and adenosine monophosphate (AMP). As extracellular ATP is a strong promoter of inflammation, monoclonal antibodies (mAbs) blocking CD39 are utilized therapeutically in the field of immune-oncology. Though anti-CD39 mAbs are highly specific for their target, they lack deep penetration into the dense tissue of solid tumors, due to their large size. To overcome this limitation, we generated and characterized nanobodies that targeted and blocked human CD39. From cDNA-immunized alpacas we selected 16 clones from seven nanobody families that bind to two distinct epitopes of human CD39. Among these, clone SB24 inhibited the enzymatic activity of CD39. Of note, SB24 blocked ATP degradation by both soluble and cell surface CD39 as a 15kD monomeric nanobody. Dimerization via fusion to an immunoglobulin Fc portion further increased the blocking potency of SB24 on CD39-transfected HEK cells. Finally, we confirmed the CD39 blocking properties of SB24 on human PBMCs. In summary, SB24 provides a new small biological antagonist of human CD39 with potential application in cancer therapy.

## 1 Introduction

Upon tissue damage and inflammation, adenosine triphosphate (ATP) is released into the extracellular space, where it acts as proinflammatory mediator (1). Extracellular ATP serves as ligand for members of the purinergic P2X receptor family (P2X1-P2X7). In particular, P2X7 is known for its role in inflammation and the release of mature interleukin-1 beta (IL-1β) (2). The amount of extracellular ATP is tightly regulated by extracellular enzymes such as the ecto-nucleotidase CD39. Structurally, human CD39 consists of 510 amino acids (aa), starting with a 16aa intracellular N-terminus, followed by a 21aa transmembrane domain, a 441aa large extracellular domain, containing the enzymatically active site, a second 21aa transmembrane domain and an 11aa intracellular C-terminus. CD39 is expressed on the surface of cells from various tissues, including blood vessel endothelial cells, platelets, cells of the innate and adaptive immune system and various tumor tissues (3, 4). CD39 converts extracellular ATP to adenosine diphosphate (ADP) and can further hydrolyze ADP to adenosine monophosphate (AMP). CD73, another ecto-nucleotidase, further converts AMP to adenosine, which can exert anti-inflammatory effects via P1 receptors, such as the A2a receptor, on immune cells (5). Therefore, the abundance and activity of CD39 and CD73 control the balance of pro-inflammatory ATP versus anti-inflammatory adenosine. It has become clear that manipulation of the CD39/CD73 axis can impact the course and/or outcome of several pathophysiological events, such as autoimmune diseases, infections, ischemia-reperfusion injuries, and cancer (3). In particular, blocking the enzymatic activity of CD39 in the setting of cancer is considered a promising approach to boost anti-cancer immunity (6–8). Small molecule inhibitors of CD39, such as ARL67156, as well as antagonistic anti-CD39 antibodies have been developed (8–12). Clinical trials using anti-CD39 mAbs TTX-030 (NCT04306900, NCT06119217) and ES002023 (NCT05075564) in cancer therapy are ongoing (www.clinicaltrials.gov).

Therapeutic antibodies represent the spearhead of modern immunomodulatory therapy for treating autoimmune diseases and cancer. As biologicals, therapeutic antibodies exhibit good pharmacological properties and are highly specific to their target. Yet, due to their large size of approximately 150 kDa, tissue penetration by conventional therapeutic antibodies is rather poor, making it difficult e.g. to reach their target antigen deep within a solid tumor. As conventional antibodies bind their cognate antigen via the variable domain of the light- (VL) and the heavy-chain (VH), the ground breaking discovery of heavy-chain-only antibodies (hcAbs) in camelids by the group of Raymund Hamers (13) paved the way for the engineering of novel small single-domain antibodies. These, 15 kDa small antigen-binding variable domains of the heavy-chain of heavy-chain-only antibodies (termed VHH or nanobodies) can be obtained from immunized camelids such as llamas or alpacas. Using phage display technology, the amino acid sequence of antigen-binding nanobodies can be determined, and nanobodies can be recombinantly produced e.g. in eukaryotic cells via transfection with a nanobody-encoding plasmid. Due to their small size of 15 kDa, nanobodies exhibit better tissue penetration than conventional antibodies (14). However, their small size leads also to the rapid renal excretion after *in vivo* application. To overcome this, Fc-nanobody fusion proteins or trimeric constructs containing an anti-albumin nanobody combined with two target-specific nanobodies have been generated (15). Further, in comparison to conventional antibodies, nanobodies have a longer complementary determining region three (CDR3), which allows them to bind epitopes in deep molecular pockets (16, 17). This feature makes them well suited for targeting and inhibiting enzymes in the extracellular space or on the cell surface (18).

Here, we report the identification and characterization of CD39-specific nanobodies from cDNA-immunized alpacas. Among the identified CD39-specific binders, clone SB24 showed potent CD39-antagonizing properties.

## 2 Methods

### 2.1 Immunization of alpacas

Two alpacas were immunized by gene gun with gold particles conjugated with huCD39-pCMVSport6 essentially as described previously (19–21). The immunization procedure was repeated on days 21, 42, and 64. On day 85, a final protein boost was performed with human CD39-transfected HEK293-T cells. Peripheral blood was drawn on days 70 and 91 and RNA was prepared from peripheral blood mononuclear cells (PBMCs). The immunization procedure was performed by Eurogentech (Belgium) according to approved local and national animal ethics protocols.

### 2.2 Generation of human CD39 stably transfected HEK cells

HEK293-T cells were cultured in a T75 cell culture flask and were transfected when cell confluence reached 70%-80%. Transfection was performed with 5 µg linearized and 5 µg circular huCD39-pCMVsport6 plasmid using JetPEI (Polyplus) according to the manufacture’s protocol. After two days, cells were harvested and stained with anti-human CD39 (clone A1, BioLegend) monoclonal antibody for cell-sorting (FACS Aria-IIIu Sorter, BD Biosciences). The fraction of cells highly expressing CD39 was isolated and the sorting procedure was repeated four times until more than 95% HEK293-T cells showed a stable high expression of human CD39.

### 2.3 Immunofluorescence staining using alpaca immune serum

CHO cells were cultured in a T75 cell culture flask and were passaged onto a 96 well plate before transfection. Transient transfection of CHO cells was performed using polyethylimidine (Sigma) and 10 µg of the huCD39-pCMVsport6 plasmid as per manufacturer’s instructions. 24h after transfection, CHO cells were gently washed once with warm phosphate‐buffered saline (PBS) and were incubated for 10 min in PBS containing 2% paraformaldehyde. Cells were washed with 200 µl PBS containing 0.02% sodium azide and incubated for 60 min at 4°C with alpaca immune serum diluted 1:600 in DMEM containing 10% fetal calf serum (FCS). Cells were washed twice with PBS and incubated for 20 min at RT with PE‐conjugated anti‐llama IgG (Abcam, ab72485) (diluted 1:200 in DMEM/FCS). Cells were washed twice with PBS and analyzed with an EVOS fluorescence microscope (Thermo Fisher Scientific) equipped with a digital camera.

### 2.4 Phage display

The VHH immune repertoire was PCR-amplified from cDNA obtained by reverse transcription of PBMC RNA. During the PCR amplification, SfiI and NotI restriction sites were introduced to the end of each VHH sequence. The VHH repertoire and phagemid pHEN2 were digested by SfiI and NotI restriction enzymes (NEB) and ligated by T4 ligase (NEB). Ligation products were used to transform TG1 cells (Lucigen), which were plated on LB agar containing carbenicillin (Thermo Fisher) at a final concentration of 100 μg/ml. Colonies on agar plates were scraped and incubated in 2×YT medium (containing carbenicillin + 20% glycerol + 2% glucose) at room temperature for 1 h. Bacterial cells were pelleted by centrifugation at 4600 rpm (Centrifuge 5810R, Eppendorf) at 4°C for 10 min. The cells were resuspended with 2×YT medium containing 20% glycerol, and stored at -80°C as bacterial library. The bacterial library was inoculated and cultured in 2×YT medium (containing carbenicillin + 2% glucose) at 37°C until OD600 reached 0.5. Bacteria were superinfected with M13K07 helper phage (BioLab) and incubated for 30 min at 37°C and 150 rpm shaking. The cultures were centrifuged for 10 min at 4°C and 4600 rpm. The bacteria pellet was resuspended in 2×YT medium containing carbenicillin and kanamycin in order to select TG1 cells co-transfected with phagemid and helper phage. Phages were produced and released into the medium during 6 h of incubation at 28°C and 220 rpm shaking. The cultures were centrifugation for 10 min at 4°C and 4600 rpm, the supernatant was added to pre-chilled PEG/NaCl buffer (Merck) and incubated on ice overnight. Phages were pelleted through centrifugation for 10 min at 4°C and 4600 rpm. The phage pellet was resuspended in 1x PBS (Gibco) and transferred into a new 1.5 ml tube with PEG/NaCl buffer. This tube was centrifuged for 5 min at 4°C and 13,000 rpm, and the supernatant was transferred to a new 1.5 ml tube with PEG/NaCl buffer. The centrifugation and transfer steps of the 1.5 ml tube were repeated 4-5 times until no pellet was visible. The purified phage library was obtained and stored at 4°C.

For panning, the phage library was incubated in blocking buffer [DMEM (1x) + GlutaMax (Gibco) + 10% FCS (Sigma) + 1% BSA (Sigma)] in a falcon tube for 1h at room temperature to minimize non-specific binding to polystyrene. Subsequently, the phage library was mixed with untransfected HEK293-T cell suspension and incubated for 1h at 4°C to remove non-specific binding to surface proteins on HEK293-T cells. The cell suspension was centrifuged for 10 min at 4°C and 4600rpm and the supernatant was added to human CD39 transfected HEK293-T cell suspension and incubated for 1h at room temperature on a vibration shaker. Then, the cell suspension was centrifuged for 10 min at 4°C and 4600rpm, to harvest the phage-bound CD39-transfected HEK cells. The cell pellet was washed 15-20 times with blocking buffer and finally resuspended in 1x PBS. HEK cell suspension was centrifuged at 4°C and 2500 rpm for 5 min and resuspended in 1x trypsin (Gibco) to eluted phages from the HEK cells. After 15 min incubation at room temperature, the phage suspension was added to a new 1.5ml tube pre-coated with AEBSF (100mM, Merck). The phage containing supernatant was centrifuged at 13000 rpm and 4°C for 5min.

After panning, the eluted phages were used to transfect TG1 cells. Transfected TG1 cells were plated on 2×YT agar containing carbenicillin and 2% glucose and incubated overnight at 37°C. Single colonies on plate were taken to extract plasmid (Miniprep Kit, Qiagen) and sequenced with primer LMB3 (5’-CAGGAAACAGCTATGAC-3’) (Eurofins). All colonies were scraped and further stored in 2×YT medium, 20% glycerol at -80°C as enriched bacterial library.

### 2.5 Recombinant production of nanobodies

VHH sequences were PCR-amplified from individual phagemid pHEN2, adding PciI and NotI restriction sites to 5’ and 3’ ends, respectively. VHH sequences were digested with PciI and NotI restriction enzymes (NEB) for 2 h at 37°C. The pCSE2.5_HIS expression vector and pCSE2.5_Fc expression vector (kindly provided by Thomas Schirrmann, Braunschweig, Germany (22), containing the coding sequence for six histidine and the Fc domain of rabbit or human IgG downstream of a NotI restriction site respectively (20), were digested with NcoI and NotI restriction enzymes (NEB). NcoI and PciI enzymes are isocaudomers and generate compatible overhangs. The digested VHH fragments were ligated with pCSE2.5_HIS and pCSE2.5_Fc vector backbone for 6His-tagged Nb monomer and Fc-Nb fusion protein production respectively, and the ligation products were used to transform XL1 blue E. coli for recombinant plasmid purification (Miniprep Kit, Qiagen).

To produce recombinant VHH- rabbit IgG hcAbs (Nb-rbFc) or monomeric His-tagged nanobodies, purified recombinant plasmid was used to transfect HEK293-6E cells using JetPEI. One day after transfection, feeding medium [FreeStyle™ 293 Expression Medium (Gibco) + 20% tryptone (Difco)] was added. Five days later, cell suspension was centrifuged for 10 min at 4600 rpm and 4°C and the supernatant containing Nb-rbFc was harvested and filtered with a Steriflip (Merck). Nb-rbFc were purified by Protein A affinity chromatography and monomeric His-tagged nanobodies were purified by immobilized metal ion affinity chromatography using Ni-NTA sepharose.

### 2.6 Octet Red96 off-rate screening

Off-rate screening of nanobodies were performed using an Octet Red96 (Fortebio). Supernatants containing Nb-rbFc were bound to Protein A Biosensors (ForteBio) and incubated first in assay buffer for 120 s. Loaded biosensors were then incubated with recombinantly produced CD39 ectodomains at 50 ng/µl. Binding responses were monitored for 300 s (association) followed by 600 s incubation with assay buffer (dissociation). Protein A biosensors were regenerated by Glycin pH 2.5 and neutralized by assay buffer, before biosensors were again loaded using supernatants containing Nb-rbFc. Visualization and calculation of dissociation constants (K_D_) were performed using Prism (Graphpad).

### 2.7 CellTiterGlo assay

Human CD39 stably transfected HEK293-T cells or recombinantly produced human CD39 ectodomains were preincubated with either Nb-rbFc containing supernatants (for screening) or purified nanobodies of indicated concentrations for 30 min at RT. ATP was added to a final concentration of 50 – 100 µM and the mixture was incubated at room temperature for 30 min. The plate was centrifuged for 5 min at 4°C and 1410 rpm and 50 µl supernatant was transferred to a new white polystyrene 96-well plate. Subsequently, 50 µl CTG reagent (CellTiter-Glo^®^ Luminescent Cell Viability Assay Kit, Promega) was added per well. After 10 min, luminescence was measured on a luminometer (Perkin Elmer). When performing functional assay human CD39 specific Nbs, 140 µl human CD39 transfected HEK293-T cell suspension were incubated with 10 µl nanobody supernatant on ice for 30 min before adding ATP.

### 2.8 High-performance liquid chromatography measurements

The blocking capacity of SB24 on the ATPase activity of recombinant human CD39 or CD39-expressing cells was analyzed by assessing its effect on the degradation of 1, *N*^6^-etheno-ATP (eATP). For this, 0.2 × 10^6^ PBMCs (stimulated for six days with 0.5 µg/mL αCD3 to increase CD39 expression on T cells) or 15 ng recombinant human CD39 were incubated with SB24 hIgG LALAPG (100 µg/mL) for 15 min at 37°C. 1 µM eATP was added to the samples and incubated further for 30 min (37°C). After the incubation, cells were removed (450 × *g*, 5 min, 4°C), and all samples were passed through 10 kDa size exclusion filters (10,000 × *g*, 10 min, 4°C, Pall Corporation), and stored at -20°C until HPLC analysis. The analysis was performed by reversed-phase HPLC on a 1260 Infinity II system (Agilent Technologies). To quantify nucleotides in the sample, different amounts of etheno-nucleotides (Biolog) were measured. The separation was performed on a 250 mm × 4.6 mm Luna C8 column (Phenomenex). The mobile phase was composed of HPLC buffer A (20 mM KH_2_PO_4_, pH 6.0) and HPLC buffer B (50% buffer A and 50% methanol) with the following gradient: 0.0 min (0.0% buffer B), 5.0 min (0.0% buffer B), 27.5 min (100.0% buffer B), 30.0 min (100.0% buffer B), 32.0 min (0.0% buffer B), 43.0 min (0.0% buffer B). The injection volume was 100 µL and the flow rate was 0.8 mL/min. The temperature of the column compartment was 20°C and the autosampler was kept at 10°C. The signals were detected by the fluorescence detector (excitation 275 nm and emission 410 nm) of the system. Peak integration was performed using OpenLab CDS software (Agilent Technologies).

### 2.9 Human material

For analysis of leukocytes, we used whole blood samples from healthy volunteer visiting the University Medical Center Hamburg-Eppendorf (UKE) or buffy coats from the Blood Bank at the UKE. All procedures for collection were approved by the local ethics committee (Ethik-Kommission der Ärztekammer Hamburg. PV5139 for samples from healthy donors) and informed consent was obtained from all donors or legal representatives.

### 2.10 Isolation of peripheral blood mononuclear cells

Peripheral blood mononuclear cells (PBMCs) were isolated from blood or buffy coats by gradient centrifugation using Biocoll as per manufacturer’s instructions (Biochrom, Germany, Berlin).

### 2.11 Flow cytometric analyses

Human CD39 stably transfected HEK293-T cells were resuspended in FACS buffer and incubated with individual Nb-rbFc at 4°C for 30 min. Cells were washed and stained with APC-conjugated anti-rabbit IgG (H+L) secondary antibody (BioLegend) at 4°C for 30 min. For staining of human primary leukocytes, peripheral blood (100 µl) was incubated with an antibody cocktail of fluorochrome-conjugated mAbs for 30 min, and indicated AlexaFluor647-conjugated anti-CD39 Nbs were added separately. This was followed by erythrocyte lysis with 1 ml of Lysing Solution for 10 min followed by two washing steps with PBS. After washing, the cell suspension was measured on FACS Celesta Flow Cytometer (BD Biosciences) and the results were analyzed with FlowJo v10 (BD Biosciences). For staining, the following monoclonal antibodies (mAs) were used: anti-CD8 BV421, anti-CD45 BV510, anti-CD3 BV605 anti-CD56 BV650, anti-HLA-DR BV785, anti-CD16 AlexaFluor488, anti-CD73 PE, anti-CD14 PerCPCy5.5, anti-CD39 PECy7, anti-CD20 AlexaFluor700, CD4 APC-Cy7, anti-CD39 Nb-hcAbs in AlexaFluor647.

## 3 Results

### 3.1 Isolation of CD39-specific nanobodies

**Figure 1:**
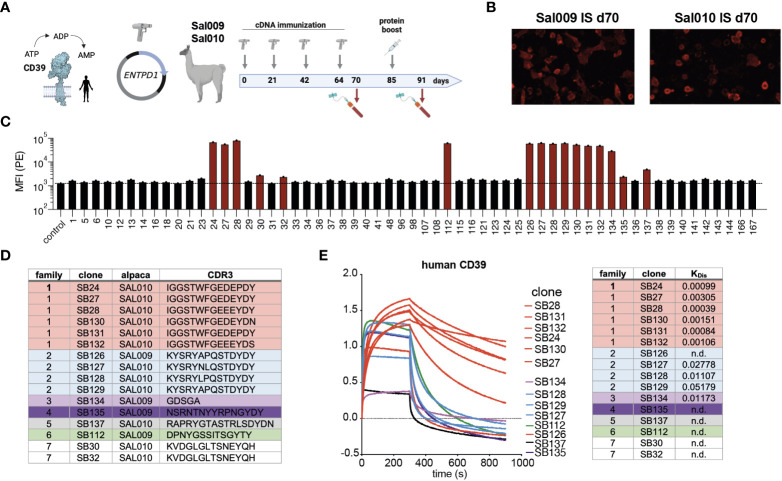
Nanobody discovery campaign results in seven different families with different binding modalities. **(A)** CD39 is an ecto-enzyme that degrades extracellular ATP into ADP and AMP. Two alpacas (SAL009 and SAL010) were immunized by cDNA immunization and a final boost with transfected HEK cells. **(B)** Immune serum obtained at d70 from both alpacas contain antibodies binding to huCD39-transfected HEK cells, as shown by immunofluorescence staining on huCD39 expressing cells. **(C)** Screening for CD39-specific nanobodies by binding to CD39 transfected HEK cells by flow cytometry. Nb-rabbit IgG hcAbs were incubated with huCD39 HEK cells and bound hcAbs were detected by PE-conjugated anti-rabbit-IgG secondary antibodies. **(D)** Grouping of Nb families according to the CDR3 region. **(E)** Binding profiles of selected nanobodies to recombinant human CD39 and K_D_ determination using biolayer interferometry analyses. The data shown in **(C, E)** are results from one out of two independently performed experiments.

VHH domains of camelid heavy-chain antibodies often bind to molecular cavities, e.g. the active site of an enzyme (23). In order to generate a biological inhibitor of the human ecto-enzyme CD39 we immunized two alpacas. As CD39 is a double-span transmembrane protein we used a cDNA immunization strategy (**Figure 1A**) by gene gun for priming on day 1 and boosting on days 21, 42 and 64 (21). Blood was taken on day 70 and was analyzed for the presence of CD39-specific antibodies. Indeed, fluorescence microscopy analyses on CD39-transfected HEK cells revealed that both alpacas had CD39-specific antibodies in their serum (**Figure 1B**). A final boost was performed by injection of CD39-transfected HEK cells on day 85. We next isolated mRNA from the peripheral blood cells obtained from the final bleeding of the immunized alpaca at day 91, generated cDNA and PCR-amplified the VHH encoding regions. These were cloned into the pHEN2 phagemid vector in order to perform phage display. In parallel to phage display screening, we cloned the VHH library in the pCSE vector in front of the hinge, CH2 and CH3 domains of rabbit IgG to directly clone and produce Nb-rb IgG heavy-chain antibodies (Nb-hcAbs) in the HEK6E expression system. Candidates obtained from direct cloning and phage display screening were sequenced and expressed as Nb-hcAbs, incubated with CD39 transfected HEK cells and bound Nb-hcAbs were detected by anti-rabbit-IgG PE-conjugated polyclonal F(ab)_2_ fragments using flow cytometry (**Figure 1C**). We identified three members of one VHH family (family 1) comprising the clones SB24, SB27 and SB28 by direct cloning. Phage display screening allowed the isolation of additional CD39-specific clones. Here, we identified three more members of family 1 (SB130, SB131 and SB132) and six additional CD39-specific nanobody families: family 2 comprising clones SB126, SB127, SB128 and SB129, families 3-6 represent four single clone families (SB134, SB 135, SB137, SB112) and family 7 containing the clones SB30 and SB32 (**Figure 1D**). These clones showed moderate to strong signals in flow cytometry. We next determined the off-rates of selected Nbs using Biolayer Interferometry. For this, Nb-hcAbs were bound to protein A biosensors and the dissociation constant K_Dis_ of recombinant human CD39 ectodomain was measured. Here, Nbs of family 1 demonstrated good binding properties, with SB28, SB24 and SB131 exhibiting off-rates (K_D_) below 0.001. In contrast, members of families 2-7 exhibited faster off-rates between 0.01 and 0.05 (**Figure 1E**). In summary, of seven Nb families selected from two alpacas immunized with human CD39, members of family 1 exhibited the best binding properties towards huCD39.

### 3.2 The selected CD39-specific nanobodies bind to two distinct epitopes on CD39

**Figure 2:**
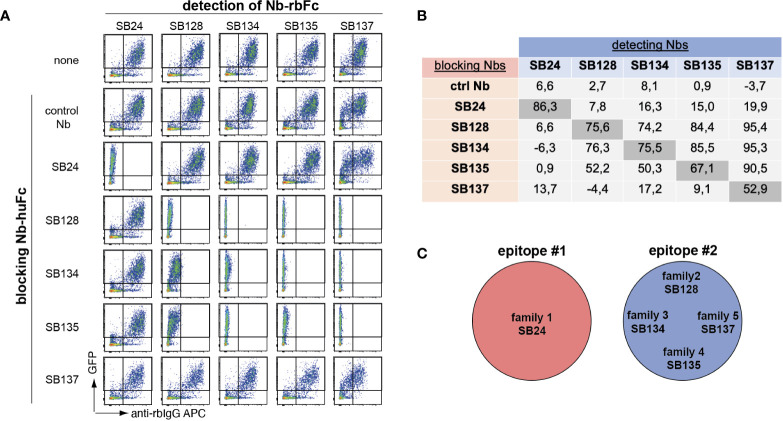
Epitope mapping of human CD39-specific nanobodies reveals coverage of two different epitopes. **(A)** HEK cells co-transfected with GFP and human CD39 were preincubated with anti-human CD39 nanobodies fused to the Fc region of human IgG (Nb-huFc), washed and incubated with anti-human CD39 nanobodies fused to the Fc region of rabbit IgG (Nb-rbFc). Binding of anti-human CD39 Nb-rbFc was detected via APC-conjugated anti-rabbit-IgG F(ab)_2_ fragments using flow cytometry. **(B)** Cross-blockade capacity was calculated as ratio of APC MFI in the presence of a blocking Nb to the APC MFI in the absence of a blocking Nb x 100 (0 = no blockade, 100 = full blockade, negative value = enhanced binding of the detection Nb in the presence of the first Nb). **(C)** Definition of two binding epitopes of anti-huCD39 Nbs. The data shown in **(A, B)** are results from one out of three independently performed experiments.

We next performed cross-blockade experiments to map the binding epitopes of the different nanobody families. For this we selected one member each of families 1-5 and expressed them as Nb-human-IgG and Nb-rabbit-IgG hcAbs. CD39-transfected HEK cells were incubated with individual Nb-hu IgG hcAbs to occupy their binding epitope. Cells were then washed and incubated with Nb-rb IgG hcAbs and bound hcAbs were was measured using PE-conjugated anti-rb-IgG F(ab)_2_ fragments and flow cytometry (**Figure 2A**). We quantified the extent of epitope blockade by calculating the ratio of the mean fluorescence intensity (MFI) of the detecting nanobody in the presence or absence of the blocking anti-CD39 Nb. As expected, pre-incubation with a control Nb-hu hcAb did not substantially affect binding of the Nb-rb hcAb (epitope blockade between –3.7 and +8.1%) (**Figure 2B**). Further, preincubation and detection with the same clone led to a pronounced blockade of binding of the respective Nb-rb hcAb (epitope blockade between 52.9 and 86.3%). Blocking with SB24 of family 1 did not substantially affect binding of the tested members of families 2-5. In contrast, blocking with SB128 of family 2 blocked binding of SB134, SB135 and SB137 of family 3-5 and vice versa, suggesting that family 1 binds a distinct epitope compared to families 2-5, which seem to share a common epitope (**Figure 2C**). Insufficient blockade using SB137 hu hcAb is likely attributed to the low affinity of SB137 (see **Figure 1E**).

### 3.3 Clone SB24 is a potent antagonist of human CD39

**Figure 3:**
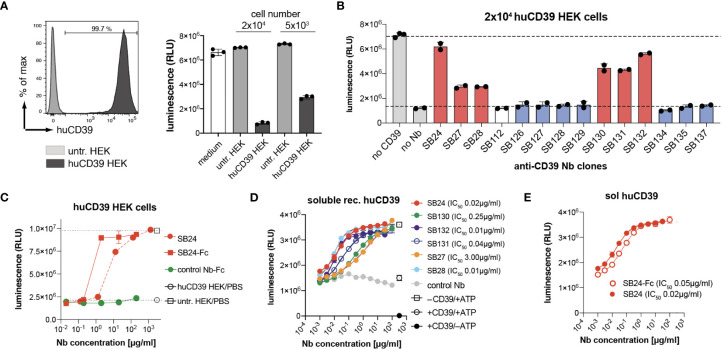
Nanobodies from family 1 are potent CD39 antagonists. **(A)** CD39 stably expressing HEK cells (20,000 or 5,000) were incubated with 100 µM ATP for 30 min. ATP derived luminescence after incubation was measured using the CellTiter-Glo assay. **(B)** 20,000 CD39-transfected HEK cells were preincubated with a saturating concentration of Nb-rabbit IgG hcAb, followed by incubation with 100µM ATP for 30 min. ATP levels after incubation were analyzed by CellTiter-Glo. Nbs binding epitope 1 are shown in red, Nbs binding epitope 2 are shown in blue. **(C)** Comparison of the capacity of monomeric SB24 and dimeric SB24 rb hcAb (SB24-Fc) to block CD39 on HEK cells using the CellTiter-Glo assay. **(D)** Monomeric Nbs were incubated with recombinant human CD39. ATP was added and ATP degradation as measured via CellTiter-Glo assay. **(E)** Comparison of the capacity of SB24 monomer and dimeric SB24 rb hcAb (SB24-Fc) to block recombinant human CD39 using the CellTiter-Glo assay. The data shown in **(A–E)** are results from one out of two independently performed experiments.

In order to evaluate the ability of the selected nanobodies to block CD39 enzymatic activity, we first established a HEK cell line that stably overexpresses human CD39. These cells were then tested for their ability to degrade extracellular ATP. For this we utilized the luciferase-based CellTiterGlo assay that generates luminescence dependent on the amount of extracellular ATP. We incubated 2 × 10^4^ and 5 × 10^3^ CD39-transfected HEK cells with 100 µM ATP and measured luminescence after 30 min of incubation. As controls, we incubated untransfected HEK cells or medium without cells with 100 µM ATP. The results show that ATP levels were reduced in a cell number dependent manner by CD39 expressing HEK cells but not by untransfected HEK cells (**Figure 3A**). Next, we pre-incubated huCD39 HEK cells with Nb-hcAbs, added 100 µM ATP and measured luminescence after 30 min. All six members of family 1 that target epitope one (red) antagonize CD39 enzymatic activity, with SB24 reaching almost full blockade (**Figure 3B**). All other tested clones of families 2-6 did not reduce the ATP-hydrolyzing enzymatic activity of CD39-transfected HEK cells. We next compared the impact of dimerization on the capacity of SB24 to block CD39 enzyme activity. For this we pre-incubated huCD39 HEK cells with increasing amounts of SB24 as monomeric nanobody or as dimeric Nb-rabbit IgG hcAbs (**Figure 3C**). The results show that dimerization increases the blocking capacity of SB24, reaching maximum blocking capacity at 2 µg/ml. Comparable results were obtained when using 130 µg/ml of the SB24 monomer. CD39 can also occur in a non-cell-bound form, e.g. on tumor or regulatory T cell derived extracellular vesicles (24, 25) and by phosphodiesterase 3-induced shedding (26). Therefore, we next analyzed the ability of the selected nanobodies to block the enzymatic activity of recombinant human CD39. First, we confirmed that recombinantly expressed CD39 was enzymatically active, as it reduced the ATP-dependent luminescence when compared to samples that contained only ATP (**Figure 3D**). We next incubated recombinant CD39 with increasing concentrations of family 1 nanobodies before adding ATP. Here, we observed a potent blockade of soluble huCD39 by SB24, SB28, SB131 and SB132 with an IC50 of 0.01 - 0.04 µg/ml. SB27 and SB130 showed a slightly reduced blocking potency with IC50 values of 0.25 - 3 µg/ml. Interestingly, dimeric SB24 rabbit IgG hcAb had a similar blocking potency toward recombinant CD39 as the SB24 monomer (**Figure 3E**).

### 3.4 SB24 binds to human immune cells and inhibits ATP degradation by PBMCs

**Figure 4:**
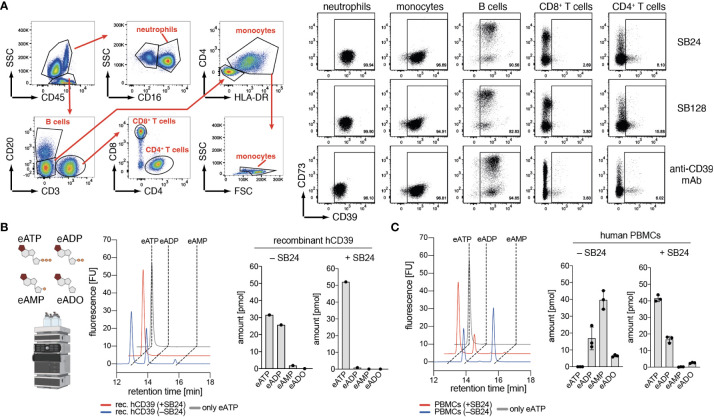
Nanobody SB24 binds to and inhibits the enzymatic activity of CD39 on human immune cells. **(A)** Flow cytometric analyses of the binding of fluorescently labeled Nb-hcAbs SB24 and SB128 to human PBMC compared to that of a conventional monoclonal antibody. **(B)** Recombinant CD39 was pre-incubated with or without SB24 (100 µg/ml) before addition of etheno-ATP (eATP, 1 µM). Breakdown of eATP into eADP and eAMP was monitored by HPLC. **(C)** Activated human PBMCs (n = 3) were incubated with or without SB24 (100 µg/ml) and eATP (1 µM) was added. Breakdown of eATP into eADP and eAMP was monitored by HPLC. The data shown in **(A, B)** are results from one out of two independently performed experiments. The data shown in **(C)** was a single experiment performed with PBMCs with from three different donors.

CD39 is expressed on a variety of human immune cells. Therefore, we tested binding of fluorochrome-conjugated-conjugated Nb-rb hcAbs SB24 (family 1) and SB128 (family 2) to primary human immune cells from peripheral blood by flow cytometry, using a commercially available monoclonal antibody (mAb) against human CD39 as control. The results show moderate expression of CD39 by neutrophils, monocytes and B cells but little if any expression by CD4^+^ and CD8^+^ T cells. Overall, the binding pattern of SB24-hcAb, SB128-hcAb and the anti-CD39 mAb were comparable, demonstrating that both Nb-hcAbs are alternatives to probe CD39 expression by flow cytometry analyses (**Figure 4A**). As CD39 can generate both, ADP and AMP from ATP, we next analyzed the impact of SB24 on the generation of both breakdown products. For this we used the fluorescent ATP analog etheno-ATP (eATP), which can be degraded by human CD39 into eADP, eAMP and, in the presence of CD73, further to etheno-adenosine (eADO). The amount of these etheno-adenine nucleotides can be quantified using high performance liquid chromatography (HPLC) (**Figure 4B**). We first tested the impact of SB24 on the enzymatic activity of soluble recombinant huCD39. Here, we observed that soluble huCD39 mainly degrades eATP to eADP, while producing only minimal amounts of eAMP. The production of both products was completely blocked in the presence of SB24. Finally, we investigated SB24-mediated blockade of ATP-degradation by human peripheral blood mononuclear cells (PBMCs) (**Figure 4C**). In the absence of SB24, PBMCs converted almost all eATP to eADP and, to a large extent, also to eAMP. In the presence of SB24 most of the eATP was preserved and only a small amount of eADP was generated. Of note, no eAMP was generated in the presence of SB24. In summary, SB24 reduces the generation of extracellular ADP and AMP from eATP by both, soluble and cell surface CD39 and thereby limits the availability of AMP as the substrate for CD73 to generate potentially immune suppressive adenosine.

## 4 Discussion

CD39 is an ecto-nucleotidase that hydrolyzes extracellular ATP to ADP and AMP. It thereby plays a pivotal role in regulating both, the level the pro-inflammatory extracellular ATP as well as the level of extracellular AMP, the substrate for the ecto-enzyme CD73 that generates anti-inflammatory adenosine. Drugs targeting CD39 enzyme activity are of interest in immuno oncology as they help to maintain a pro-inflammatory environment thereby favoring anti-tumor immune responses that would otherwise be blunted by adenosine. In the present study we report the generation of new nanobody-based inhibitors of the human CD39. We identified seven Nb families covering two distinct binding epitopes. Members of family 1 bound to epitope 1, whereas Nbs of all other families bound to epitope 2. Family 1 contains clones with the strongest binding capacity. In contrast to other families, members of family 1 inhibited the enzyme activity of huCD39, both as a recombinant soluble enzyme as well as a cell surface enzyme of human PBMCs.

So far only small molecule- or conventional antibody-based CD39 inhibitors have been developed. With CD39-targeting nanobodies we introduced a new class of CD39-blocking biologicals that exhibit several advantages compared to CD39-antagonizing antibodies. First, they are much smaller than conventional antibodies, which allows them to penetrate tissues more easily (27). Further, they exhibit high solubility, due to their hydrophilic framework region 2 (FR2) (28). Nanobodies can be produced recombinantly at high yield in eukaryotic expression systems, often tolerate temperatures up to 75°C (29), are resistance to proteases and endure pH changes. These attributes make nanobodies an interesting option for diagnostics and therapeutics.

An advantage of nanobodies is the ease with which they can be reformatted to suit specific desired applications, e.g. fused to tailor-made effector domains or as bispecific reagents to either recruit effector cells or to target the nanobody to a specific cell population. This could enable targeting of CD39-blocking nanobodies to tumor-infiltrating T cells or CD39-expressing tumor cells while preserving CD39 enzyme activity in other tissues. This is beneficial because CD39 degrades extracellular ADP, which serves as ligand for the P2Y12 receptor expressed on platelets leading to thrombocyte aggregation and thrombosis. As CD39 is constitutively expressed on endothelial cells in order to maintain blood fluidity (30), using a cell-specific anti-CD39 Nb approach would preserve endothelial CD39 enzymatic activity while blocking CD39 on cells relevant for anti-tumor therapy.

In our study we found that SB24 as Fc-fusion protein was considerably more effective in blocking cell-bound CD39 activity when compared to the SB24 monomer. In contrast, the soluble form of CD39 was similarly blocked by SB24-Fc or SB24 monomers. Indeed, there are different factors that can influence the effectiveness of the blockade. Cell-bound antigens might be more difficult to bind since some epitopes might be facing towards the plasma membrane or are masked by surrounding proteins. This might explain the overall higher amount of nanobodies needed to block cell membrane CD39 compared to soluble CD39. Additionally, the soluble CD39 lacks both transmembrane domains, which might alter the stability of the recombinant protein and render it more prone to inactivation. A second factor is avidity. For soluble monomeric CD39 the interaction to SB24 will be monomeric, regardless of the SB24 construct. When CD39 is expressed on the cell surface, SB24-Fc can bind in a dimeric interaction, whereas SB24 monomer will still bind in a monomeric kinetic, lacking the avidity-effect by dimerization.

The CD39-specific nanobodies identified in this study cover two distinct binding epitopes. The antagonistic family 1 members bind to epitope #1 whereas all other tested nanobodies bind to a non-overlapping epitope #2. The identification of clones against two distinct binding epitopes allows the generation of biparatopic Nb constructs (31), allowing the binding of a dimeric Nb construct to a single CD39 molecule. It needs to be evaluated whether this strategy improves the inhibitory action of SB24 fused to e.g. SB134 or another clone. For other targets, it has been already demonstrated that biparatopic nanobodies can have enhanced target binding or modulatory capacity. For example biparatopic nanobodies against the RBD of SARS-COV2 protect mice from lethal challenge with SARS-CoV-2 variants (32); biparatopic Nb-hcAbs against CD38 show enhanced complement activation (33), CXCR4-specific biparatopic nanobodies can mobilize stem cells and exhibit superior anti-HIV activity compared to nanobodies binding a single epitope (34), and CART cells based on a biparatopic Nb-CART cells show better therapeutic efficacy than ones based on conventional scFvs (35). Future studies will show whether combining two distinct-paratope-binding CD39-specific nanobodies improves CD39 antagonism and if a biparatopic construct can be combined with cell/tissue targeting nanobodies.

A major field of application for CD39-antagonizing biologicals is cancer therapy. Preclinical studies revealed that blocking mouse CD39 by an anti-CD39 mAb (clone B66) in a MC38 tumor mouse model drives extracellular ATP- and inflammasome-driven anti-tumor immunity with the potential to overcome anti-PD1 resistance (36). Another preclinical study demonstrated that the human CD39 antagonistic mAb IPH5201 unleashed immune responses in combination with chemotherapy, when evaluated in tumor models in human CD39 knock in mice. Of note, we evaluated SB24 already in a previous study and could demonstrate that SB24 was able to increase NK-92 cell-mediated cytotoxicity and act synergistically with TIGIT and A2AR blockade (37). Consequently, CD39-targeting antibodies entered clinical trials (6). SRF617 is a CD39-specific human IgG4 antibody that, similarly to SB24, inhibits the ATPase activity of human CD39 (38). SRF617 is being tested on patients with advanced solid tumors in a phase 1 dose-escalation study (NCT04336098) to establish safety as monotherapy and in combination with other drugs. Preliminary data demonstrate that SRF617 is well-tolerated at doses that sustain target occupancy throughout the dosing interval, supporting the initiation of expansion cohorts (NCT04336098). Another clinical trial is currently evaluating the CD39-specific antibody TTX-030 in combination with immunotherapy and/or chemotherapy in patients with advanced cancers (NCT04306900). Regarding Nb SB24, the next logical step is the evaluation in preclinical tumor mouse models. Here, the modular nature and small size of the nanobody could be advantageous: bi-specific nanobodies targeting CD39 on cancer cells and cancer-specific antigens e.g. epidermal growth factor receptor (EGFR) could establish an ATP focus within solid tumors, as a small dimeric, bi-specific nanobody construct of ~30 kDa is predicted to penetrate deep into solid tumors. CD39-antagonistic nanobodies hold promise for boosting anti-tumor immunity, especially in combination with other cancer therapy approaches.

## Data availability statement

The raw data supporting the conclusions of this article will be made available by the authors, without undue reservation.

## Ethics statement

The studies involving humans were approved by Ethik-Kommission der Ärztekammer Hamburg (PV5139 for samples from healthy donors). The studies were conducted in accordance with the local legislation and institutional requirements. The participants provided their written informed consent to participate in this study.

## Author contributions

SM: Conceptualization, Writing – original draft, Data curation, Investigation, Methodology, Writing – review & editing. YD: Investigation, Methodology, Writing – review & editing, Validation. JH: Methodology, Writing – review & editing. BA: Methodology, Writing – review & editing. DW: Methodology, Writing – review & editing. RW: Methodology, Validation, Writing – review & editing. ET: Conceptualization, Funding acquisition, Supervision, Writing – review & editing. AR: Methodology, Validation, Writing – review & editing. AG: Funding acquisition, Supervision, Writing – review & editing. FH: Conceptualization, Funding acquisition, Supervision, Writing – review & editing. TM: Conceptualization, Funding acquisition, Supervision, Writing – review & editing. FK-N: Conceptualization, Funding acquisition, Supervision, Writing – review & editing. BR: Conceptualization, Funding acquisition, Visualization, Writing – original draft, Writing – review & editing.
